# Pre-diagnostic colonoscopies reduce cancer mortality - results from linked population-based data in South Australia

**DOI:** 10.1186/s12885-019-6092-4

**Published:** 2019-08-29

**Authors:** Ming Li, Ian Olver, Dorothy Keefe, Carol Holden, Dan Worthley, Timothy Price, Christos Karapetis, Caroline Miller, Kate Powell, Dianne Buranyi-Trevarton, Kellie Fusco, David Roder

**Affiliations:** 1 Cancer Research Institute, University of South Australia, Adelaide, Australia; 2 SA Cancer Service, South Australian Department for Health and Wellbeing, Adelaide, Australia; 3 South Australia Health and Medical Research Institute, Adelaide, Australia; 4 Clinical Oncology Research Unit, The Queen Elizabeth Hospital, Woodville, Australia; 5 Flinders Medical Centre, Flinders University, Adelaide, Australia

**Keywords:** Colonoscopy history, Colorectal cancer death, Competing risk analysis, South Australia, Linked inpatient and medical benefits schedule data

## Abstract

**Background:**

To investigate the association between pre-diagnostic colonoscopy and colorectal cancer mortality in South Australia.

**Methods:**

Colonoscopy histories were obtained for colorectal cancer patients diagnosed in 2003–2013 using linked Medical Benefits Schedule (MBS) claims, hospital-inpatient and cancer-registry data. Colonoscopy histories included the year of colonoscopy, numbers of examinations, and the time from first colonoscopy to diagnosis. Histories of multiple exposures to colonoscopies, and exposures of greater than a year from initial colonoscopy to diagnosis, were regarded as indicators of screening or surveillance activity. Colonoscopies occurring within one year of diagnosis were regarded as more likely to be a response to cancer symptoms than those occurring > 1 year before diagnosis. Associations between colonoscopy history and post-diagnostic survival were analysed using sub-hazard ratios (SHRs) from competing risk regression adjusted for socio-demographic and cancer characteristics.

**Results:**

Having pre-diagnostic colonoscopy was associated with an unadjusted reduction in risk of colorectal cancer death of 17% (SHR: 0.83, 95% CI 0.78–0.89). After adjusting for time period and sociodemographic characteristics, the risk of colorectal cancer death reduced by 17% for one pre-diagnostic colonoscopy examination; 27% for two pre-diagnostic colonoscopy examinations; and 45% for three or more pre-diagnostic colonoscopy examinations. Those with a time of over one year from first colonoscopy in the study window to diagnosis, when compared with less than one year, had a 17% lower risk of colorectal cancer death in this adjusted analysis. These reductions were substantially reduced or eliminated when also adjusting for less advanced stage.

**Conclusions:**

Pre-diagnostic colonoscopy, and more so, multiple colonoscopies and first colonoscopy occurring over one year from initial colonoscopy to diagnosis, were associated with longer survival post diagnosis. This was largely explained by less advanced cancer stage at the time of diagnosis.

**Electronic supplementary material:**

The online version of this article (10.1186/s12885-019-6092-4) contains supplementary material, which is available to authorized users.

## Background

Australian cancer registry data indicate that colorectal cancer has the third highest recorded incidence of cancer after prostate and breast cancer [1]. Efforts to improve outcomes of colorectal cancer have been beneficial with survival gains attributed to advances in adjuvant therapy, increased surgical specialisation, and earlier detection [2, 3].

The National Bowel Cancer Screening Program (NBCSP) commenced in 2006 with a staggered introduction occurring by age during 2006–2020 [2]. A data linkage study found that bowel cancers diagnosed through the NBCSP were more likely to present at an earlier stage at diagnosis than other bowel cancers [2].

The NBCSP aims to reduce morbidity and mortality from bowel cancer through biennial screening by faecal occult blood testing (FOBT) of the population aged 50–74 years, and with follow-up colonoscopy of FOBT- positive cases [2–4]. The NBCSP follows Australian Clinical Practice Guidelines for the Prevention, Early Detection and Management of Colorectal Cancer, which is endorsed by the National Health and Medical Research Council [4].

Despite an absence of randomized trial evidence of effects of colonoscopy screening on colorectal cancer mortality, the US Preventive Services Taskforce supports use of FOBT with colonoscopy as a primary screening test [5]. Other evidence of mortality reductions exists from earlier detection. Trial results indicate that screening by FOBT can reduce colorectal cancer mortality [6] and evidence exists that sigmoidoscopy examinations can reduce colorectal cancer mortality [7–10].

This is the first study using linked cancer registry and administrative data to explore effects of pre-diagnostic colonoscopy screening on colorectal cancer mortality in South Australia and whether mortality benefits are occurring as expected from the US Preventive Services Taskforce recommendations [5]. Its focus is evaluation of translation of research evidence into practice rather than contributing to the scientific basis for pre-diagnostic colonoscopy.

While earlier linked data have shown sociodemographic disparities in survival and surgical mortality for colorectal cancer [11, 12], research into effects of colonoscopy surveillance on colorectal cancer outcomes has been limited in Australia. This study uses colorectal cancer data for South Australia to examine associations between pre-diagnostic colonoscopy and survival.

## Methods

### Data sources and linkage

Data on colorectal cancers (ICD10 C18–20) diagnosed between January 2003 and December 2013 were extracted from the South Australian Cancer Registry (SACR), which collects under legal mandate data on all invasive colorectal cancer diagnoses in South Australian residents [11, 12]. Data coverage is virtually complete and data fields collected include cancer topography and morphology, age at diagnosis, date of diagnosis, country of birth, Indigenous status, postcode-derived relative socioeconomic disadvantage and geographic remoteness, local health network of residence, and death date and cause [11–16].

To these data were added Australian Clinico-Pathological Staging (ACPS) data derived from SACR records [17]. Pre-treatment clinical staging was used, which included evidence from MDT reports, diagnostic biopsies and imaging reports (including data from ultrasound examinations, MRI of the pelvis, CT scans, and PET), and reports of endoscopic and other physical examination [17].

While screening participation and FOBT outcome data collected by NBCSP are close to complete, reporting of NBCSP colonoscopies is not legally mandated and is incomplete [2]. We have therefore used integrated hospital inpatient data and Medical Benefits Schedule (MBS) claims to gain a better estimate of the use of pre-diagnostic colonoscopies.

Hospital inpatient data for 2002–2013 were obtained from the Integrated South Australian Activity Collection (ISAAC), including year of admission and clinical procedure codes. MBS claims data on endoscopies were also extracted for 2000–2013, including dates of services and item numbers. Inpatient data included treatment and procedures for all hospitalized patients while MBS covered tests and examinations by doctors to diagnose and treat illnesses for out-of-hospital services. Population coverage is virtually complete. MBS is a universal health insurance scheme that covers colonoscopy examinations undertaken in the private health sector whereas hospital inpatient data cover colonoscopies undertaken on inpatients.

Probabilistic and deterministic linkage methods were used to link SACR, ISAAC inpatient and MBS claims data. Data linkage between the SACR and ISAAC collections was undertaken by SANT DataLink through probabilistic matching using name, sex, date of birth and address. Data linkage between these data and MBS claims data was undertaken through the Australian Institute of Health and Welfare (AIHW), using a study linkage key also derived from names, sex, dates of birth, and addresses. The process of data linkage followed the principle of separating patient identifiers from clinical content data to protect privacy.

The study was approved by the South Australia Human Research Ethics Committee (HREC/16/SAH/6), the University of South Australia’s Human Research Ethics Committee (0000036128), and the Australian Institute of Health and Welfare Human Research Ethics Committee (EO2016/4/317).

### Data coding and classification

ISAAC procedure codes used for colonoscopy were 3,209,000, 3,209,001, 3,209,002 and 3,209,300, whereas the corresponding MBS codes were 32,090 for fibreoptic colonoscopy of the colon beyond the hepatic flexure without biopsy; and 32,093 for colonoscopy examinations with the removal of polyps. History of pre-diagnostic colonoscopy in the study window was classified by: (a) number of colonoscopy examinations (none, one, two, > 3), and (b) time from colonoscopy to cancer diagnosis (< 1 year, > 1 year). For the purposes of this study methodology, we assumed that colonoscopies undertaken in response to cancer symptoms were more likely to have occurred in the one-year period immediately preceding diagnosis, whereas screening and surveillance colonoscopies were more likely to have occurred prior to this one-year period [4].

Age at diagnosis was classified as < 60, 60–69 or 70+ years; country of birth as country/region using the Standard Australia Classification of Countries [14]; socioeconomic disadvantage using the Index of Relative Socioeconomic Disadvantage (SEIFA 2006) based on Statistical Local Areas grouped into quintiles (the first quintile being the most disadvantage and the 5th quintile being the least disadvantaged) [15]; and remoteness of residence based on Statistical Local Areas aggregated according to the 2006 Australian Standard Geographical Classification (ASGC) as major city, inner regional, outer regional, remote, or very remote [16]. Local health networks (LHNs) of residence were determined by data custodians from residential postcodes and classified as Country Health South Australia, Central Adelaide, Southern Adelaide, and Northern Adelaide.

Degree of spread of cancer at diagnosis was obtained from Australian Clinico-Pathological Staging (ACPS) and summarised as localised, regional or distant metastases using Tumour-lymph Node- Metastasised (TNM) criteria which were broadly equivalent to SEER summary staging criteria [17].

### Statistical analysis

Pre-diagnostic colonoscopy examinations were analysed by population and cancer characteristic using the chi-square test or other non-parametric tests depending on variable distribution. Deaths were classified: as due to colorectal cancer or another cause, based on SACR records; and by time to death (irrespective of cause) between diagnosis and death. For those alive at the end of the study follow-up period (December 31st, 2013), the follow-up period was the duration between diagnosis and this end date.

The association of colonoscopy with risk of colorectal cancer-specific death was analysed using competing risk regression models (STATA module “stcrreg”). Deaths from causes other than colorectal cancer were regarded as the competing risk. The association was firstly adjusted for population characteristics and colonoscopy time period (Model 1); and then in addition for cancer stage (Model 2). Risk of colorectal cancer mortality was analysed by colonoscopy history (i.e., number of colonoscopies, period and follow-up time from first colonoscopy to diagnosis). Reference categories for the dummy variables used in analyses were the first categories listed in Tables 1, 2, 3.

**Table 1:** Population and cancer characteristics by pre-diagnostic colonoscopy among colorectal cancer patients registered in 2003–2013, South Australia (*n* = 12,906)

	No colonoscopy *n* = 8194	Colonoscopy *n* = 4712	Total *n* = 12,906	*P* value
Mean age (SD) at diagnosis (years)	69.4 (13.5)	71.5 (12.0)	70.2 (13.0)	< 0.001
Age at diagnosis (years)				< 0.001**
< 60	1843 (22.5%)	764 (16.2%)	2607 (20.0%)	
60–69	2025 (24.7%)	1067 (22.7%)	3092 (24.0%)	
70–79	2191 (26.8%)	1554 (33.0%)	3745 (29.0%)	
> 80	2129 (26.0%)	1326 (28.2%)	3455 (26.8%)	
Sex				0.043*
Male	4365 (54.7%)	2447 (52.8%)	6812 (54.0%)	
Female	3621 (45.3%)	2188 (47.1%)	5809 (46.0%)	
Country of birth				< 0.001*
Australia & NZ	5486 (68.9%)	3206 (70.2%)	8692 (69.4%)	
Europe	1931 (24.3%)	1153 (25.3%)	3084 (24.6%)	
North Africa/Middle East	18 (0.2%)	16 (0.4%)	34 (0.3%)	
Asia & Pacific Islands	114 (1.5%)	52 (1.2%)	166 (1.4%)	
Americas	31 (0.4%)	8 (0.2%)	39 (0.3%)	
Africa (other)	370 (4.7%)	130 (2.9%)	500 (4.0%)	
Indigenous				0.100*
No	7596 (99.8%)	4476 (99.6%)	12,072 (99.7%)	
Yes	19 (0.3%)	19 (0.4%)	38 (0.3%)	
Remoteness				< 0.001**
Major city	6022 (73.5%)	3174 (67.4%)	9196 (71.3%)	
Inner regional	893 (10.9%)	508 (10.8%)	1401 (10.9%)	
Outer regional and remote	1278 (15.6%)	1029 (21.8%)	2307 (17.9%)	
SES disadvantage quintile				< 0.001**
1st (most disadvantage)	1884 (23.0%)	1327 (28.2%)	3211 (24.9%)	
2nd	1813 (22.1%)	1058 (22.5%)	2871 (22.3%)	
3rd	1545 (18.9)	936 (19.9%)	2481 (19.3%)	
4th	1615 (19.7%)	809 (17.2%)	2424 (18.8%)	
5th (least disadvantage)	1332 (16.3%)	572 (12.2%)	1904 (14.8%)	
Local Health Network				< 0.001*
Country Health SA	2424 (29.6%)	1689 (35.9%)	4113 (31.9%)	
Central Adelaide	2507 (30.6%)	1269 (26.9%)	3776 (29.3%)	
Northern Adelaide	1628 (19.9%)	872 (18.5%)	2500 (19.4%)	
Southern Adelaide	1634 (19.9%)	880 (18.7%)	2514 (19.5%)	
Median (IQR) of years survived on Dec 31st 2013	2.7 (0.9–5.5)	3.0 (1.1–6.3)	2.8 (1.0–5.7)	< 0.001
Cancer staging (max spread)				< 0.001**
Local	2001 (42.9%)	1479 (49.8%)	3480 (45.6%)	
Regional	1556 (33.4%)	897 (30.2%)	2453 (32.1%)	
Distant	1108 (23.8%)	595 (20.0%)	1703 (22.3%)	
% cancer-specific survival by time from diagnosis				< 0.001***
At 1 year	84.5%	86.8%	85.5%	
At 3 year	71.0%	74.6%	72.3%	
At 5 year	64.6%	69.4%	66.0%	

*p from Fisher’s exact or Chi-square test; **p from Mann-Whitney U test; ***p from log-rank test for equality of survivor functions; note - cell totals < overall totals due to missing records

**Table 2:** Characteristics associated with colorectal cancer mortality post diagnosis and colonoscopy history (deaths by December 31st, 2013, registered in 2003–2013, South Australia (*n* = 12,906)

Characteristics	Colorectal cancer death *n* = 3771	Other death *n* = 1621	Alive *n* = 7514	Unadjusted SHR (95% CI) *
Mean age (SD) at diagnosis (years)	72.2 (13.2)	78.3 (10.0)	67.4 (12.6)	1.13 (1.11–1.16)
Age at diagnosis (years)				
< 60	652 (17.3%)	68 (4.2%)	1887 (25.1%)	1.00
60–69	781 (20.8%)	193 (11.9%)	2118 (28.2%)	1.00 (0.91–1.11)
70–79	1056 (28.0%)	495 (30.5%)	2194 (29.2%)	1.13 (1.03–1.25)
> 80	1280 (34.0%)	865 (53.4%)	1310 (17.5%)	1.70 (1.55–1.87)
Sex				
Male	2014 (53.6%)	917 (56.6%)	3881 (53.6%)	1.00
Female	1745 (46.4)	703 (43.4%)	3361 (46.4%)	1.03 (0.97–1.10)
Country of birth				
Australia & NZ	2599 (71.1%)	1113 (71.1%)	4980 (68.3%)	1.0
Europe	957 (26.2%)	427 (27.3%)	1700 (23.3%)	1.03 (0.96–1.11)
North Africa/ Middle East	10 (0.3%)	2 (0.1%)	22 (0.3%)	0.99 (0.54–1.82)
Asia and Pacific Islands	47 (1.2%)	15 (1.0%)	106 (1.5%)	0.87 (0.66–1.15)
Americas	14 (0.4%)	2 (0.1%)	23 (0.3%)	1.35 (0.80–2.28)
Africa (other)	32 (0.9%)	7 (0.5%)	461 (6.3%)	0.66 (0.46–0.95)
Indigenous				
No	3653 (99.5%)	1576 (99.9%)	6843 (99.7%)	1.00
Yes	17 (0.5%)	2 (0.1%)	19 (0.3%)	1.85 (1.12–3.05)
Remoteness				
Major city	2663 (70.6%)	1199 (74.0%)	5334 (71.0%)	1.00
Inner regional	386 (10.2%)	156 (9.6%)	859 (11.4%)	0.95 (0.85–1.05)
Outer regional and remote	722 (19.2%)	266 (16.4%)	1319 (17.6%)	1.11 (1.02–1.20)
SES disadvantage quintile				
1st (most disadvantage)	1020 (27.1%)	406 (25.1%)	1785 (23.8%)	1.00
2nd	838 (22.3%)	346 (21.4%)	1687 (22.5%)	0.92 (0.84–1.00)
3rd	689 (18.3%)	365 (22.5%)	1427 (19.0%)	0.84 (0.77–0.93)
4th	699 (18.6%)	283 (17.5%)	1442 (19.2%)	0.89 (0.81–0.98)
5th (least disadvantage)	519 (13.8%)	220 (13.6%)	1165 (15.5%)	0.82 (0.73–0.91)
Local Health Network code				
Country Health SA	1211 (32.1%)	474 (29.2%)	2428 (32.3%)	1.00
Central Adelaide	1122 (29.8%)	527 (32.5%)	2127 (28.3%)	1.00 (0.92–1.09)
Northern Adelaide	755 (20.0%)	273 (16.8%)	1472 (19.6%)	1.04 (0.95–1.14)
Southern Adelaide	683 (18.1%)	347 (21.4%)	1484 (19.8%)	0.90 (0.82–0.98)
Years survived on Dec 31st 2013 Median (IQR)	2.8 (0.9–5.1)	2.8 (0.9–5.1)	4.4 (1.9–7.2)	
Cancer staging (max spread)				
Local	439 (18.1%)	547 (59.6.%)	2494 (58.2%)	1.00
Regional	783 (32.2%)	277 (30.2%)	1393 (32.5%)	3.05 (2.72–3.42)
Distant	1208 (49.7%)	94 (10.2%)	401 (9.4%)	11.41 (10.24–12.71)
Pre-diagnosis colonoscopy				
No	2505 (66.4%)	899 (55.5%)	4790 (63.8%)	1.00
Yes	1266 (33.6%)	722 (44.5%)	2724 (36.3%)	0.83 (0.78–0.89)
Follow-up time from initial colonoscopy to diagnosis				
< 1 year	883 (69.8%)	529 (73.3%)	1577 (57.9%)	1.00
> =1 year	383 (30.3%)	193 (26.7%)	1147 (42.1%)	0.82 (0.73–0.93)
Pre-diagnostic colonoscopy examinations				
One	1023 (80.8%)	560 (77.6%)	1991 (73.1%)	1.00
Two	179 (14.1%)	119 (16.5%)	446 (16.4%)	0.90 (0.77–1.05)
> = 3	64 (5.1%)	43 (6.0%)	287 (10.5%)	0.63 (0.49–0.81)

*SHR (sub Hazard ratio) from competing risk survival analysis with death other than colorectal cancer as competing risk in the univariate model; cell totals < overall totals due to missing records

**Table 3:** Adjusted sub-hazard ratios (95% CI) of colorectal cancer death with history of pre-diagnostic colonoscopy among patients diagnosed in 2003–2013*

	No. patient	Person time (person year)	No. Event	Model 1	Model 2
Pre-diagnostic colonoscopy examinations					
0	8194	28,653.08	2505	1.00	
1	3574	14,305.83	1023	0.83 (0.77–0.90)	0.85 (0.79–0.91)
2	744	2600.52	179	0.73 (0.63–0.89)	0.81 (0.70–0.95)
> = 3	394	1226.19	64	0.55 (0.43–0.70)	0.68 (0.53–0.86)
Time from initial colonoscopy to diagnosis					
< 1 year	2989	12,481.72	883	1.00	1.00
> = 1 year	1723	5650.82	383	0.83 (0.73–0.94)	0.97 (0.85–1.10)

*Sub hazard ratios (SHR) from competing risk survival regression model

Analyses were conducted using STATA 14 (STATACorp, College Station, Texas, USA), with statistical significance level set as *p* < 0.05. All the analyses were based on complete case data.

## Results

### Descriptive features

Linked data for the 12,906 colorectal cancer patients diagnosed in 2003–2013 indicated the following descriptive features: a mean age of 70.2 years (SD 13.0); 56% aged 70+ years at diagnosis; 54% male; 94% born in Australia, New Zealand or Europe; 0.3% Indigenous (Aboriginal or Torres Strait Islander); 71% resident in a major city (metropolitan Adelaide); a quarter from the most socio-economically disadvantaged areas (quintile 1); and 46% of those with a recorded stage having localized disease (Table 1).

### Pre-diagnostic colonoscopy

Of the 12,906 colorectal cancer patients, 37% had pre-diagnostic colonoscopy. Compared to those without this history, they were older at diagnosis; more likely to have been born in Australia, New Zealand or Europe; and more likely to live in outer regional or remote, and more socioeconomically disadvantaged areas. They were also more likely to have a localized cancer and to experience a higher survival at five years post diagnosis (Table 1).

Of those having a pre-diagnostic colonoscopy, 76% had one colonoscopy, 16% had two, and 8% had three or more colonoscopies (a maximum of 11). They comprised 3048 patients having 3583 colonoscopies recorded in hospital data, and 1963 patients having 2969 colonoscopies recorded in MBS claims (note: 299 of these patients had colonoscopies recorded in both the hospital and MBS data).

The mean time from initial colonoscopy to cancer diagnosis was 1.5 years, with an interquartile range of 0–2.5 years. Of the 4712 having a pre-diagnostic colonoscopy, 63% were diagnosed with colorectal cancer within one year of this initial colonoscopy examination and 37% more than one year after this initial pre-diagnostic colonoscopy.

Compared with those having only one colonoscopy, those having more than one were significantly (*p* < 0.05) more likely: (a) to be aged 60–79 years; (b) to have been born in Australia or New Zealand; (c) to live in socioeconomically advantaged areas; (d) to be diagnosed more than one year after initial colonoscopy; (e) to be diagnosed with localized as opposed to more advanced tumours; and (f) to have longer survival (Additional file 1: Table S1).

### Cancer survival

Of the 12,906 patients with colorectal cancer, 29% died from their cancer and 13% of other causes in the follow-up period to December 31st, 2013. The mean survival time from diagnosis was 2.8 years (IQR 1.0–5.7).

Unadjusted sub-hazards ratios (using other deaths as competing risks) were: (a) higher for ages 70+ years at diagnosis than < 60 years; (b) lower for patients born in Africa (other) than Australia and New Zealand; (c) higher for Indigenous than non-Indigenous patients; (d) higher for residents living in outer regional and remote than major city areas; (e) lower for residents of less disadvantaged areas; (f) lower for localized cancers; and (g) lower for place of residence in the Southern Adelaide than the Country Health South Australia Network (Table 2).

Patients having a pre-diagnostic colonoscopy were at a lower unadjusted risk of colorectal cancer death (SHR 0.83, 95% CI 0.78–0.89) compared with those not having a colonoscopy (Table 2). Among those having a pre-diagnostic colonoscopy, the unadjusted risk was lower in patients having a pre-diagnosis colonoscopy more than one year before diagnosis (SHR 0.82 (0.73–0.93), and compared with those having one pre-diagnostic colonoscopy, potentially lower in those having two (SHR 0.90 (0.77–1.05)) and lower in those having 3+ pre-diagnostic colonoscopies (SHR 0.63 (0.49–0.81)) (Table 2).

When adjusting for socio-demographic characteristics and initial colonoscopy year, the risk of colorectal cancer death reduced by: (a) 17% when having one colonoscopy (SHR 0.83, 95% CI 0.77–0.90); (b) 27% when having two colonoscopies (SHR 0.73, 95% CI 0.63–0.89); and (c) 45% when having three or more colonoscopies (SHR 0.55, 95% CI 0.43–0.70) (Table 3: Model 1). The risk also reduced in this analysis when the time from initial colonoscopy to diagnosis was one year or more (SHR 0.83, 95% CI 0.73–0.94) compared with less than one year (Table 3: Model 1). Adjusting in addition for stage reduced the scale of reductions in risk of colorectal cancer death to 32% for three or more colonoscopies (SHR 0.68, 95% CI 0.53–0.86) and largely eliminated the reduction in risk where the initial colonoscopy occurred more than one year pre-diagnosis (SHR 0.97, 95% CI 0.85–1.10) (Table 3: Model 2).

Other factors found in the final full model to be associated with an increased risk of colorectal cancer death included: older age (SHR for aged 70–79 years of 1.36, 95% CI 1.22–1.47, and for aged 80 + years of 1.92, 95% CI 1.75–2.12 compared with < 60 years); being Indigenous (SHR 1.57, 95% CI 1.07–2.31); and having an advanced cancer stage when compared with localized stage (SHR for regional stage of 3.05, 95% CI 2.71–3.43 and for distant stage of 11.16, 95% CI 9.99–12.46). By comparison, socioeconomic advantage was associated with a lower risk of colorectal cancer death (SHR for the most advantaged of 0.85, 95% CI 0.76–0.95 compared with most disadvantaged).

Model 1: adjusted for age, sex, country of birth, Indigenous status, residential remoteness, socioeconomic status, local health network, initial colonoscopy year; Model 2: adjusted for the same characteristics as Model 1, plus stage.

Notably sex, country of birth, residential remoteness, and local health district were no longer associated with risk of colorectal cancer death in the multivariate competing risk regression analysis (p > 0.050). Also, the association between pre-diagnostic colonoscopy and colorectal cancer death in the fully adjusted model was not different among subgroups classified by age at diagnosis, Indigenous status and socio-economic status (*p* > 0.050).

## Discussion

Results indicate that having a pre-diagnostic colonoscopy was associated with a 17% lower risk of colorectal cancer death. After adjusting for sociodemographic and cancer characteristics, the risk of colorectal cancer death reduced step-wise with increasing numbers of colonoscopy examinations to 45% for three or more compared with no colonoscopy. Also, a 17% reduction applied when the time to diagnosis from initial colonoscopy was > 1 year compared with < 1 year.

Stage appeared to mediate much of these reductions such that adjusting in addition for stage: [1] reduced the scale of reduction with increased number of colonoscopies; and [2] largely eliminated the increased reduction associated with a longer time from colonoscopy to diagnosis.

Our results indicate a protective benefit of pre-diagnostic colonoscopy which is consistent with results from the USA Nurses’ Health Study and Health Professionals Follow-up [18] and the more recent case-control study of Veterans Affairs patients [19]. Effect sizes were larger in the USA data, potentially reflecting differences in reasons for colonoscopies. We consider that the use of colonoscopy in this South Australian study would have included more symptomatic cases leading to a smaller reduction in risk of colorectal from pre-diagnostic colonoscopy. Other factors that could have affected the reduction in risk included sample selection and follow-up period [20–22].

Our results support the conclusion that pre-diagnostic colonoscopy in South Australia lowers the risk of colorectal cancer death, largely by effecting an earlier stage at diagnosis. The association of a lower risk with multiple colonoscopy examinations and longer duration from initial colonoscopy to diagnosis (1+ years) is suggestive of a protective benefit from colonoscopy used in response to positive FOBT screening results in the average risk population or to surveillance of high-risk patients drawn from primary health care settings. By comparison, colonoscopy in response to colorectal cancer symptoms likely would have occurred closer to diagnosis (i.e., within a year).

Results support the importance of adherence to Australian clinical guidelines on early detection of colorectal cancer [4]. To reduce colorectal cancer mortality further, participation in the National Bowel Screening Program should be increased through promotion of awareness among primary health care practitioners and the Australian public. Present levels of FOBT screening are low, with a 2-year participation rate of only 41% between January 2015 and December 2016. Moreover, of those having a positive FOBT result, only 68% were reported to have had follow-up diagnostic assessment [23].

Residents of remote and low socio-economic areas, and Indigenous screeners, have lower rates of colonoscopy and longer durations to colonoscopy assessment following a FOBT, despite elevations in positive FOBT results [23]. Operational research into the most effective strategies for addressing these disparities is needed.

The study also has limitations. We could not determine the purpose of colonoscopies from the direct recordings available to us through bowel screening registries, MBS and inpatient datasets and we inferred the purpose of colonoscopies from the duration from the initial colonoscopy to diagnosis, and evidence of multiple colonoscopies. We recognize that this approach would have lacked the accuracy possible though direct reporting, had it been available. Nonetheless, the data indicate a lower risk of colorectal cancer death when the initial colonoscopy was over a year ago, and where there were multiple colonoscopy examinations (both of which were considered to be indicative of screening or surveillance). The association of earlier colonoscopies with reduced deaths was largely explained by earlier stage, which we interpret as a screening or surveillance benefit.

In addition, potential for misclassification due to the limited time window from inpatient data to extract pre-diagnostic colonoscopy also was a limitation. Specifically, the time period to capture MBS-funded colonoscopy data was from 2000, which was > 2 years before the earliest diagnosis of colorectal cancer cases in the study window (2003–2013) and longer than the 2-year screening interval recommended for the national bowel cancer screening program (NBCSP). An equivalent window was not available from public hospital data for pre-diagnostic colonoscopies in 2000–2001 (i.e., a period > 1 year before the study window). Although we would expect a small proportion of colonoscopies with negative results occurring > 1 year before diagnosis to have been missed for this reason, we think it unlikely that this would have greatly affected the results. It would not have affected the size of the group with colonoscopies occurring < 1 year before diagnosis.

Another limitation was the low numbers of cases with 1+ years occurring between initial colonoscopy and diagnosis, limiting opportunities for further sub-classification of this group (note: the average duration from initial colonoscopy to diagnosis was 1.5 years, with an interquartile range of 0–2.5 years). However, we consider that 1+ years was a reasonable cut-off to separate colonoscopies that were more likely to have been be undertaken in response to cancer symptoms from those more likely to have been for screening or surveillance.

Incomplete staging data were also a key limitation. This was due to limited coverage of staging data by the electronic clinical information systems available to us. While this may have led to selection bias, we see no obvious reason for this to have occurred. Predictably these data showed a positive association of colonoscopy history with lower spread of the cancer at diagnosis. We consider that presenting these data in the context of this limitation in stage completeness is justified.

Finally, potential confounding effects on colorectal cancer mortality outcomes from differences in prevalence of comorbid conditions and related risk behaviours (including effects of smoking, alcohol drinking, physical activity, obesity, diabetes, and medication use) could not be investigated with the data available [24].

## Conclusions

Having a pre-diagnostic colonoscopy is associated with a reduced risk of colorectal cancer death in South Australia. This relationship is independent of age at diagnosis, sex, country of birth, Indigenous status, socioeconomic status, residential remoteness, local health network of residence, and initial examination period.

Among those having a pre-diagnostic colonoscopy, higher numbers of colonoscopy examinations, and longer times from first colonoscopy examination to diagnosis, are associated with a reduced risk of colorectal cancer death, irrespective of socio-demographic characteristics. This is largely due to less advanced cancers at diagnosis.

The reduced risk of colorectal cancer death with multiple colonoscopy examinations, and longer time from first colonoscopy examination, is indicative of a benefit from screening or surveillance of high-risk patients, since it would largely exclude colonoscopies undertaken close to cancer diagnosis in response to symptoms.

## Additional file

Additional file 1: **Table S1.** Population characteristics of colorectal patients in South Australia in 2003–2013 by numbers of pre-diagnostic colonoscopies (*n* = 4712). *One-way ANOVA for age at diagnosis, **Fisher’s exact chi-square tests; cell totals < overall totals due to missing records. (DOCX 18 kb)

## Data Availability

The data that support the findings of this study are available from the South Australian Department for Health and Wellbeing (South Australian Cancer Registry & Integrated South Australian Activity Collection) and the Australian Institute for Health and Welfare (Medical Benefits Schedule) but restrictions apply as conditions for data custodian and ethics approvals for the study. As a result, source data are publicly available only with approval from these bodies.

## Abbreviations

ACPS: Australian Clinico-Pathological Staging
AIHW: Australian Institute of Health and Welfare
ASGC: Australian Standard Geographical Classification
CHSA: Country Health South Australia
CI: Confidence interval
FOBT: faecal occult blood test
ICD10: International Classification of Diseases, 10th revision
IQR: Interquartile range
ISAAC: Integrated South Australian Activity Collection
LHNs: Local health networks
MBS: Medical Benefits Schedule
NBCSP: The National Bowel Cancer Screening Program
NZ: New Zealand
SACR: South Australian Cancer Registry
SD: Standard deviation
SEIFA: Socio-Economic Indexes for Areas
SHR: Sub-distribution hazard ratio
TNM: Tumour-lymph Node- Metastasised
