# Supplementary material for: Pre-diagnostic colonoscopies reduce cancer mortality - results from linked population-based data in South Australia
Source: BMC Cancer. 2019 Aug 29;19:856. doi: 10.1186/s12885-019-6092-4 (PMC6716808; doi:10.1186/s12885-019-6092-4)
Supplement: Supplementary file 1 — Table S1. Population characteristics of colorectal patients in South Australia in 2003–2013 by numbers of pre-diagnostic colonoscopies (n = 4712). *One-way ANOVA for age at diagnosis, **Fisher’s exact chi-square tests; cell totals < overall totals due to missing records. (DOCX 18 kb) [file 12885_2019_6092_MOESM1_ESM.docx]

**Table S1. Population characteristics of colorectal patients in South Australia in 2003-2013 by numbers of pre-diagnostic colonoscopies (n=4712)**

| **Characteristics** | **Once n=3574** | **Twice n=744** | **>=3 times n=394** | **P** |
| --- | --- | --- | --- | --- |
| **Mean age (SD) at diagnosis (years)** | 71.3 (12.3) | 72.5 (11.3) | 72.1 (10.2) | 0.020* |
| **Age at diagnosis (yrs.)** |  |  |  | <0.001 |
| <60 | 626 (17.5%) | 98 (13.2%) | 40 (10.2%) |  |
| 60-69 | 797 (22.4%) | 162 (21.8%) | 108 (27.4%) |  |
| 70-79 | 1159 (32.4%) | 257 (34.6%) | 138 (35.0%) |  |
| >80 | 992 (27.8%) | 226 (30.4%) | 108 (27.4%) |  |
| **Sex** |  |  |  | 0.762 |
| Male | 1862 (53.1%) | 382 (51.7%) | 203 (52.2%) |  |
| Female | 1645 (46.9%) | 357 (48.3%) | 186 (47.8%) |  |
| **Country of birth** |  |  |  | 0.041** |
| Australia and NZ | 2399 (69.3%) | 508 (70.6%) | 299 (78.1%) |  |
| Europe | 898 (25.9%) | 180 (5.0%) | 75 (19.6%) |  |
| North Africa/Middle East | 11 (0.3%) | 5 (0.7%) | 0 (0) |  |
| Asia & Pacific Islands | 44 (1.3%) | 6 (0.8%) | 2 (0.5%) |  |
| Americas | 6 (0.2%) | 2 (0.3%) | 0 (0) |  |
| Africa (other) | 104 (3.0%) | 19 (2.6%) | 7 (1.8%) |  |
| **Indigenous** |  |  |  | 0.877** |
| No | 3386 (99.6%) | 709 (99.6%) | 381 (9.7%) |  |
| Yes | 15 (0.4%) | 3 (0.4%) | 1 (0.3%) |  |
| **Remoteness** |  |  |  | 0.112 |
| Major city area | 2432 (68.1%) | 479 (64.4%) | 263 (66.8%) |  |
| Inner regional | 369 (10.3%) | 86 (11.6%) | 53 (13.5%) |  |
| Outer regional and remote | 772 (21.6%) | 179 (24.1%) | 78 (19.8%) |  |
| **SES disadvantage quintile** | |  |  | <0.001 |
| 1^st^ (most disadvantage) | 1039 (29.2%) | 219 (29.4%) | 69 (17.5%) |  |
| 2^nd^ | 790 (22.2%) | 174 (23.4%) | 94 (23.9%) |  |
| 3^rd^ | 705 (19.8%) | 143 (19.2%) | 88 (22.3%) |  |
| 4^th^ | 611 (17.1%) | 125 (16.8%) | 73 (18.5%) |  |
| 5^th^ (least disadvantage) | 419 (11.8%) | 83 (11.2%) | 70 (17.8%) |  |
| **Local Health Network code** | |  |  | 0.029 |
| Country Health SA | 1254 (35.1%) | 289 (38.8%) | 146 (37.1%) |  |
| Central Adelaide | 963 (27.0%) | 195 (26.2%) | 111 (28.2%) |  |
| Northern Adelaide | 653 (18.3%) | 152 (20.4%) | 67 (17.0%) |  |
| Southern Adelaide | 703 (19.7%) | 107 (14.4%) | 70 (17.8%) |  |
| **Cancer staging (max)** |  |  |  | 0.001 |
| Local | 1119 (47.9%) | 255 (56.8%) | 105 (57.1%)) |  |
| Regional | 734 (31.4%) | 110 (24.5%) | 53 (28.8%) |  |
| Distant | 485 (20.7%) | 84 (18.7%) | 26 (14.1%) |  |
| **Vital status at the end of 2013** | |  |  | <0.001 |
| Alive | 1991 (55.7%) | 446 (60.0%) | 287 (72.8%) |  |
| Colorectal cancer death | 1023 (28.6%) | 179 (24.1%) | 64 (16.2%) |  |
| Other deaths | 560 (15.7%) | 119 (16.0%) | 43 (10.9%) |  |
| **Diagnosed within one year after initial colonoscopy** | | |  | <0.001 |
| No | 897 (25.1%) | 464 (62.4%) | 362 (91.9%) |  |
| Yes | 2677 (74.9%) | 280 (37.6%) | 32 (8.1%) |  |

*One-way ANOVA for age at diagnosis, **Fisher’s exact chi-square tests; cell totals < overall totals due to missing records
